# Symptom burden among long-term survivors of young adult cancer: a report from the Project Milestones cohort

**DOI:** 10.1007/s11764-026-01986-7

**Published:** 2026-02-20

**Authors:** David R. Freyer, Marcie D. Haydon, Rebecca K. Kelly, Erin M. Mobley, Wendy Mack, Michael E. Roth, Kimberly A. Miller, Ann S. Hamilton, Joel E. Milam

**Affiliations:** 1 Departments of Pediatrics, Medicine, and Population and Public Health Sciences, Keck School of Medicine, University of Southern California; USC Norris Comprehensive Cancer Center; Cancer and Blood Disease Institute, Children’s Hospital Los Angeles, 4650 Sunset Boulevard, Mailstop 54, Los Angeles, CA 90027-6016 USA; 2 Population Health and Disease Prevention, University of California Irvine, Irvine, CA USA; 3 Cancer and Blood Disease Institute, Children’s Hospital Los Angeles, Los Angeles, CA USA; 4 Department of Surgery, College of Medicine Jacksonville, University of Florida, Jacksonville, FL USA; 5 Department of Population and Public Health Sciences, Keck School of Medicine, University of Southern California, Los Angeles, CA USA; 6 Department of Pediatrics, University of Texas MD Anderson Cancer Center, Houston, TX USA; 7 Department of Epidemiology and Biostatistics, University of California Irvine, Irvine, CA USA

**Keywords:** Cancer survivorship, Symptom burden, Young adult cancer, Late effects

## Abstract

**Purpose:**

Although late effects and their symptomatology are well described for childhood cancer survivors, less is known about survivors of young adult (YA) cancer. Our aim was to characterize symptom burden among long-term survivors of YA cancer.

**Methods:**

Project Milestones is a cross-sectional cohort survey study assessing benchmarks of emerging adulthood among 3–10-year cancer survivors diagnosed 21–39 years old. We analyzed responses from the first half of cohort participants to 22 questions that screened for current, clinically significant symptoms. Statistical analyses included Chi-square, Fisher exact, and negative binomial regression tests.

**Results:**

There were 1,025 evaluable participants (68.9% female; 34.2% Hispanic; median age at diagnosis and survey 31 and 39 years, respectively; and 73% ≥ 5 years post-treatment). Cancer types were reproductive (male/female, 30.4%), leukemia/lymphoma (28.2%), thyroid (13.5%), breast (10.3%), melanoma (8.9%), and colorectal (8.8%). The most-endorsed symptoms were fatigue (39.4%), altered appearance (35.8%), cognitive problems (31.7%), general pain (28.0%), sensory neuropathy (24%), and urinary incontinence (20%). Over 70% reported at least 1 symptom; one-third reported ≥ 4. In adjusted analysis, cumulative symptom count was significantly higher among participants who were female (vs. male), Hispanic (vs. non-Hispanic White), had public insurance (vs. employer-sponsored), and received chemotherapy/radiation (vs. surgery) by 44%, 21%, 49%, and 71%, respectively.

**Conclusions:**

Long-term survivors of YA cancer report a substantial burden of persistent, clinically significant symptoms.

**Implications for Cancer Survivors:**

Clinicians should emphasize the importance of sustained survivorship care and monitor for symptoms suggesting late effects. Further research is needed to understand their contributing factors and functional impact.

**Supplementary Information:**

The online version contains supplementary material available at https://doi.org/10.1007/s11764-026-01986-7.

## Introduction

Due to treatment advances, 5-year aggregate survival for adolescents and young adults (AYAs, 15–39 years old) treated for newly diagnosed cancer now exceeds 85% [1]. In the United States, approximately 90,000 AYAs are diagnosed with cancer annually and recent estimates indicate there are over 2 million long-term survivors of AYA cancer [1, 2]. Given their relatively young age, survivors of AYA cancer anticipate decades of life after treatment, which includes managing long-term health as a foundation for the life-stage goals of completing education, launching careers, achieving financial independence, establishing long-term relationships, and beginning families. Therefore, knowledge of the prevalence and types of adverse health outcomes, including chronic illness and symptomatology, is crucial for providing optimal survivorship care to this growing population.

To date, few published studies have described health outcomes among long-term survivors of AYA cancer. This is in sharp contrast to the population of young adult survivors of childhood cancer, which since the 1990 s has been the focus of several large, ongoing, longitudinal cohort studies such as the Childhood Cancer Survivor Study (CCSS), the Dutch Childhood Cancer Survivor Study, and United Kingdom Childhood Cancer Study [3–5]. Studying young adults previously treated with diverse multimodal regimens for various childhood cancers, these landmark efforts have characterized their substantial burden of physical and psychosocial late effects, including cumulative incidence, severity, and host- and treatment-related risk factors [6–9]. This research approach has led to practice-changing outcomes including implementation of clinical guidelines for risk-adapted late effects surveillance and modification of treatment regimens to reduce toxicity [10–12].

However, knowledge about the physical health of long-term survivors of AYA cancer is considerably more limited. Most reported studies are restricted to survivors of a particular cancer type or who are early in follow-up, and are unable to shed light on the status of long-term survivors of AYA cancer as a whole [13–15]. Recently, two multi-diagnosis cohorts of long-term survivors of AYA cancer have been established that help define the prevalence of late effects in this population [16, 17]. However, the burden of late or persistent symptomatology among these survivors remains poorly understood. Although the AYA HOPE Study enrolled survivors of various AYA cancers and reported significantly elevated levels of fatigue and other health problems compared with non-cancer controls, the sample was limited to early survivors recruited within 36 months of diagnosis and did not capture long-term health status [18, 19]. Thus, there remains a need for population-representative cohort studies to better understand the late effects spectrum and symptom burden experienced by long-term survivors of AYA cancer. Such information would inform an essential and richer understanding of the unmet needs and their impacts on functional outcomes and life-stage goals.

This analysis was conducted as part of Project Milestones, an in-progress population-based cohort study of developmental benchmarks of emerging adulthood among long-term survivors following treatment for 11 prototypical AYA cancers. We report survey responses from approximately the first half of 2,000 planned participants who were asked about current experiences with 22 symptoms potentially referable to treatment-related late effects. With a sizeable sample and the opportunity to contribute much-needed data while awaiting completion of the parent study, the objective of this early analysis was to describe symptom prevalence and correlates in this contemporary cohort.

## Methods

### Study design and population

Project Milestones is a population-based, cross-sectional cohort study primarily assessing developmental benchmarks of emerging adulthood among survivors of young adult (YA) cancer who were diagnosed in Southern California (Los Angeles and Orange Counties). Cases were obtained from the California Cancer Registry (CCR) and Los Angeles Cancer Surveillance Program, part of the National Cancer Institute Surveillance, Epidemiology, and End Results (SEER) Program. Inclusion criteria identified those (1) age 21–39 years at diagnosis with one of the following eleven prototypical YA cancers: acute leukemia, breast, cervix, colorectal, Hodgkin lymphoma, melanoma, non-Hodgkin lymphoma (NHL), ovary, thyroid, testis, or uterine; (2) diagnosed between 2010–2017; and (3) English or Spanish fluency. Participants with a history of relapse or a subsequent primary cancer were eligible. To adjust for the wide variability in incidence, enrollment of the most common YA cancer types (thyroid, breast, testis, melanoma, NHL, Hodgkin lymphoma, and cervical cancer) was capped to allow sample enrichment with those that are less common (colorectal, uterine, leukemia, and ovary). To adjust for the wide variability in prognosis and ensure sufficient recruitment of long-term survivors, only local/regional stages were included for melanoma, cervix, corpus, ovary, breast, and colorectal cancer, whereas all stages were included for thyroid, testis, NHL, and Hodgkin lymphoma. Participants must have been post-cytotoxic, cancer-directed treatment for at least one year per self-report. Due to smaller subsets, we combined cancers that were gynecologic (cervix, corpus uterus, and ovary) or hematologic (NHL, Hodgkin lymphoma, and leukemia) for this analysis.

### Procedure

Based on our prior work, recruitment to Project Milestones included introductory postcards and mailings with brochures describing the study, the CCR, and self-report surveys in English and Spanish [20]. Participants only (not proxies) could complete the approximately 45-min survey online, by phone, or by mail. Participants received a $30 gift card after survey completion. Procedures were approved by the California State Committee for the Protection of Human Subjects and the University of Southern California and University of California, Irvine Institutional Review Boards prior to beginning recruitment.

### Measures

#### Demographic and clinical factors

Age at diagnosis, biological sex, race/ethnicity (non-Hispanic White, non-Hispanic Black, Hispanic, Asian, and other), cancer diagnosis (site/histology, stage), and quintile of neighborhood socioeconomic status (nSES) at diagnosis were obtained from the CCR. nSES is a census-based composite score (relative to California’s statewide distribution, 1 = Lowest quintile and 5 = Highest quintile) [21, 22]. Attained age and years since diagnosis were determined at study entry. Current health insurance (employer-sponsored, public, individual, other, or uninsured) was self-reported. Using CCR and self-reported data, treatment categories were created to identify those treated with (1) surgery only, (2) chemotherapy/no radiation, (3) radiation/no chemotherapy, or (4) chemotherapy and radiation.

#### Symptom reports

Using a binary response (Yes/No), twenty-two survey items assessed whether the participant was currently experiencing clinically significant general or organ system-specific symptoms and conditions (Supplemental Fig. 1). Six of these were sex-specific (orchiectomy, erectile dysfunction, azoospermia, premature menopause, oophorectomy, and altered breasts) while the other 16 were not. Hereafter, the term “symptom” is used to denote both symptoms and conditions assessed. Items were developed by the authors based on clinical experience and were designed to capture with a single question the most salient indicator of serious dysfunction in the anatomic system or functional domain assessed, a streamlined approach necessitated by the large size of the overall Project Milestones survey, which is primarily focused on life-stage outcomes. Total symptom counts were calculated using only the 16 non-sex-specific questions and were categorized as 0, 1, 2–3, or ≥ 4 symptoms.

### Statistical analysis

This early analysis focused on the first 1,151 responders enrolled. Interim responder analyses (comparing responders to those who did not respond during the same time window) were performed using chi-square tests for available registry-provided demographic and clinical variables.

The prevalence of symptoms among respondents was examined both individually and cumulatively. In addition to frequencies, statistical analysis included chi-square, and Fisher exact tests for comparison of proportions of participants endorsing symptoms. For evaluating associations between demographic and clinical covariates and cumulative symptom count, multivariable negative binomial regression was selected to address overdispersion in symptom count (*M* = 2.54, *SD* = 2.59) [23]. Interval since diagnosis was included as the exposure variable to account for variability in time since diagnosis and treatment. Effect size was expressed as rate ratios, with coefficients indicating a percentage increase (values > 1) or decrease (values < 1) in symptom count compared with the referent. Independent variables were attained age (continuous), sex, race/ethnicity, stage of disease, and treatment type. Covariates (nSES, health insurance) were selected based on prior empirical studies [24, 25]. Cancer type was not included due to collinearity with sex (e.g., breast and reproductive cancers) and type of treatment received. Statistical significance was determined as p < 0.05 for 2-sided hypothesis tests. Analyses were conducted using Stata BE 17 (StataCorp LLC, 2021).

## Results

### Participant characteristics

A total of 4,380 cancer survivors were invited to participate in the overall Project Milestones study. At the time of this analysis, there were 1,151 responders, approximately half of the planned 2,000 participants. There were no differences between responders and non-responders (n = 3,229) in age at diagnosis, attained age, years since diagnosis, and cancer stage (Supplemental Table 1). Those who responded were more likely to be leukemia, lymphoma, thyroid, or breast cancer survivors, female, non-Hispanic White, and higher nSES. Excluding participants within 1 year of treatment (n = 91) or with missing/incomplete data (n = 35), the final analytic sample size was 1,025. Of respondents, 85% (n = 875) completed the survey online and the rest on paper (15%; n = 150); 5% (n = 53) responded in Spanish, and 1% responded in both English and Spanish (n = 13).

Participants (68.9% female, 34.2% Hispanic) had a mean age of 31 years at diagnosis (median 31, range 21–39) and a mean attained age of 39 years (median 39, range 24–52) (Table 1).

**Table 1:** Participant characteristics

Characteristics	Total (*N* = 1,025)	Male (*n* = 319)	Female (*n* = 706)
Demographics			
Age at cancer diagnosis, years			
Median (range)	31 (21–39)	31 (21–39)	32 (21–39)
Attained age, years			
Median (range)	39 (24–52)	39 (25–52)	40 (24–52)
Years since diagnosis			
Median (range)	8 (3–13)	8 (3–13)	8 (3–13)
Years since treatment, No. (%)			
< 2	58 (5.7)	25 (7.8)	33 (4.7)
2–4	200 (19.5)	60 (18.8)	140 (19.8)
5 or more	751 (73.3)	230 (72.1)	521 (73.8)
Did not answer	16 (1.6)	4 (1.3)	12 (1.7)
Biological sex, No. (%)			
Female	706 (68.9)	–	–
Male	319 (31.1)	–	–
Race or ethnicity, No. (%)			
Non-Hispanic White	506 (49.4)	160 (50.2)	346 (49.0)
Hispanic	351 (34.2)	107 (33.5)	244 (34.6)
Non-Hispanic Asian	116 (11.3)	31 (9.8)	85 (12.0)
Non-Hispanic Black	33 (3.2)	11 (3.5)	22 (3.1)
Other or unknown	19 (1.9)	10 (3.1)	9 (1.3)
Neighborhood socioeconomic status, No. (%)			
Low	166 (16.2)	50 (15.7)	116 (16.4)
Low-middle	197 (19.2)	61 (19.1)	136 (19.3)
Middle	191 (18.6)	58 (18.2)	133 (18.8)
Middle-high	247 (24.1)	90 (28.2)	157 (22.2)
High	224 (21.9)	60 (18.8)	164 (23.2)
Health insurance type, No. (%)			
Employer-sponsored	619 (60.4)	196 (61.4)	423 (59.9)
Public	164 (16.0)	47 (14.7)	117 (16.6)
Individual	88 (8.6)	21 (6.6)	67 (9.5)
Other/Unknown	107 (10.4)	42 (13.2)	65 (9.2)
No insurance	47 (4.6)	13 (4.1)	34 (4.8)
Clinical characteristics			
Cancer type, No. (%)			
Leukemia/Lymphoma^1^	289 (28.2)	138 (43.3)	151 (21.4)
Gynecologic^2^	221 (21.6)	0 (0)	221 (31.3)
Thyroid	138 (13.5)	17 (5.3)	121 (17.1)
Breast	106 (10.3)	0 (0)	106 (15.0)
Testicular	90 (8.8)	90 (28.2)	0 (0)
Colorectal	90 (8.8)	46 (14.4)	44 (6.2)
Melanoma	91 (8.9)	28 (8.8)	63 (8.9)
Cancer stage, No. (%)			
0/I	558 (54.4)	132 (41.4)	426 (60.3)
II	184 (18.0)	57 (17.9)	127 (18.0)
III	121 (11.8)	49 (15.4)	72 (10.2)
IV	47 (4.6)	24 (7.5)	23 (3.3)
Not applicable	79 (7.7)	40 (12.5)	39 (5.5)
Unknown	36 (3.5)	17 (5.3)	19 (2.7)
Treatment, No. (%)			
Surgery only	333 (32.5)	84 (26.3)	249 (35.3)
Chemotherapy, no radiation	347 (33.9)	158 (49.5)	189 (26.8)
Radiation, no chemotherapy	115 (11.2)	18 (5.6)	97 (13.7)
Chemotherapy and radiation	221 (21.6)	58 (18.2)	163 (23.1)

^1^Hodgkin lymphoma n = 107, non-Hodgkin lymphoma n = 100, Leukemia, unspecified n = 79, Other n = 3

^2^Cervical n = 82, Uterine n = 65, Ovarian n = 54, Endometrial n = 13, Female gynecologic, unspecified n = 7

Participants were an average of 8 years from diagnosis (median 8, range 3–13) and 73.3% of participants were ≥ 5 years post-treatment. The most common cancer diagnoses were leukemia/lymphoma (28.2%), gynecologic (21.6%), and thyroid (13.5%) cancers. Most participants (84.2%) had malignancies that were Stage III or less, and slightly over half (54.4%) had Stage 0/I. One-third of participants underwent surgery only (32.5%), about half had received either chemotherapy or radiation (45.1%), and a fifth received both chemotherapy and radiation (21.6%). Over half (54.0%) lived in Low to Middle nSES neighborhoods. Most (60.4%) participants had employer-provided insurance and 4.6% had no insurance.

### Prevalence of current symptoms

**Fig. 1:**
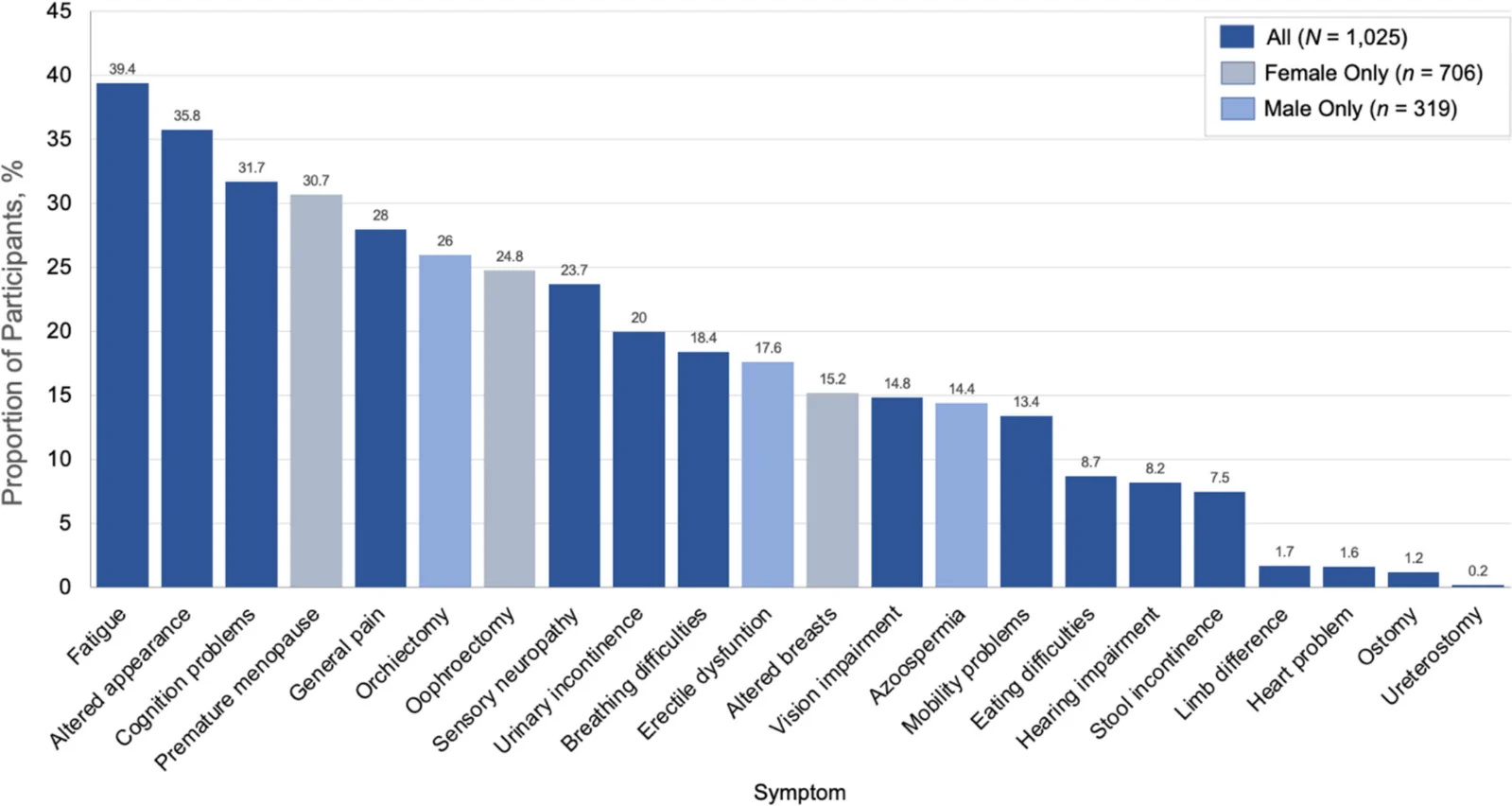
Proportion of respondents (n = 1,025) endorsing the presence of 22 current symptoms or reported conditions potentially attributable to underlying late effects resulting from prior cancer treatment. Dark blue bars represent problems applicable to all participants. Sex-specific problems are represented by grey bars (female, n = 706) and light blue bars (males, n = 319)

Fatigue was the most-endorsed symptom, affecting 39.4% of participants (Table 2, Fig. 1). Symptoms (and conditions) affecting approximately one fifth to one third of participants included altered appearance (35.8%), cognitive problems (31.7%), premature menopause (30.7%, females only), general pain (28.0%), orchiectomy (26.3%, males only), oophorectomy (24.8%, females only), sensory neuropathy (23.7%), urinary incontinence (20.0%), and difficulty breathing (18.4%).

**Table 2:** Prevalence of self-reported symptoms

	No. of Participants (%)			
Symptom	Total (*N* = 1,025)	Male (*n* = 319)	Female (*n* = 706)	p^*^
Fatigue	404 (39.4)	91 (28.5)	313 (44.3)	** < 0.001**
Altered appearance	367 (35.8)	102 (32.0)	265 (37.5)	0.086
Cognition problems	325 (31.7)	69 (21.6)	256 (36.3)	** < 0.001**
General pain	286 (28.0)	81 (25.4)	205 (29.0)	0.23
Sensory neuropathy	243 (23.7)	52 (16.3)	191 (27.1)	** < 0.001**
Urinary incontinence	205 (20.0)	22 (6.9)	183 (25.9)	** < 0.001**
Breathing difficulties	189 (18.4)	52 (16.3)	137 (19.4)	0.24
Vision impairment	152 (14.8)	27 (8.5)	125 (17.7)	** < 0.001**
Mobility problems	137 (13.4)	42 (13.2)	95 (13.5)	0.90
Eating difficulties	89 (8.7)	25 (7.8)	64 (9.1)	0.52
Hearing impairment	84 (8.2)	26 (8.2)	58 (8.2)	0.97
Stool incontinence	77 (7.5)	33 (10.3)	44 (6.2)	**0.021**
Limb difference	17 (1.7)	7 (2.2)	10 (1.4)	0.43^^^
Heart problem	16 (1.6)	7 (2.2)	9 (1.3)	0.28^^^
Ostomy	12 (1.2)	6 (1.9)	6 (0.9)	0.21^^^
Ureterostomy	2 (0.2)	1 (0.3)	1 (0.1)	0.53^^^
Sex-Specific Symptoms				
Orchiectomy	–	84 (26.3)	–	–
Erectile dysfunction	–	56 (17.6)	–	–
Azoospermia	–	46 (14.4)	–	–
Premature menopause	–	–	217 (30.7)	–
Oophorectomy	–	–	175 (24.8)	–
Altered breasts	–	–	107 (15.2)	–

^*^Chi-square tests except where indicated. ^^^Fisher’s exact test

Other notable symptoms affecting 8% to 15% of participants included impaired vision, mobility problems, eating difficulties, hearing impairment, and stool incontinence. Compared with males, females had a statistically significantly higher prevalence of fatigue, cognition problems, sensory neuropathy, urinary incontinence, and vision impairment, whereas males had a statistically significantly higher rate of stool incontinence (Table 2, Fig. 1). There were notable variations in symptom endorsement by sex (Table 2) and cancer type (Supplemental Table 2).

**Fig. 2:**
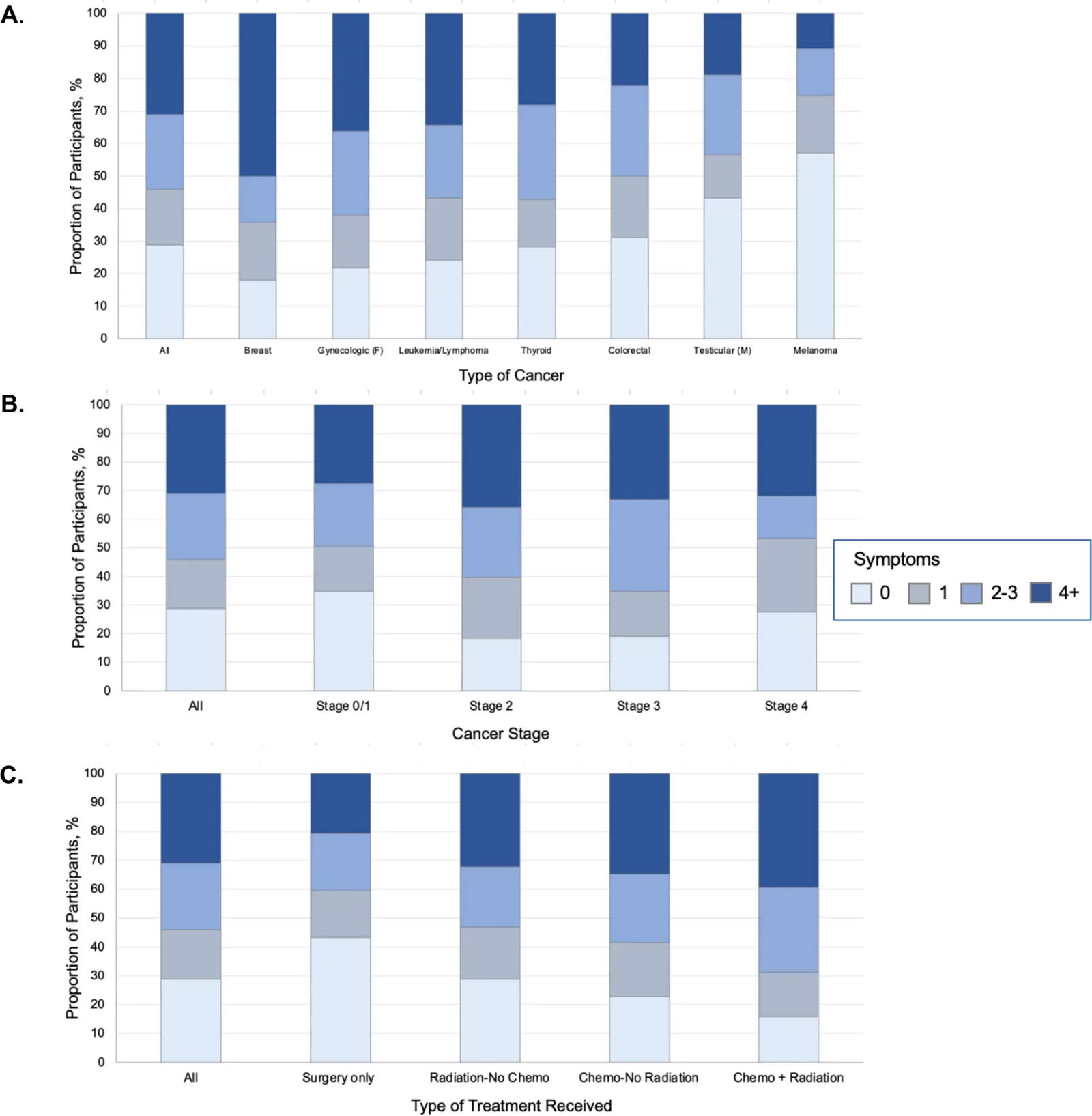
Proportion of respondents endorsing the presence of 0, 1, 2–3, or ≥ 4 current symptoms/conditions from among a list of 16 potentially attributable to underlying late effects resulting from prior cancer treatment and applicable to all participants (sex-specific symptoms/conditions excluded for this analysis). Respondents were categorized according to the number of problems endorsed and are represented by differing bar colors (light blue = 0, grey = 1, medium blue = 2–3, and dark blue = 4 or more). The proportion of respondents endorsing current problems are presented by cancer type (**A**), cancer stage (**B**), and cancer treatment received (**C**). The sample represents 1,025 participants (female, 706; male 319)

For total symptom count, 71.2% reported at least one symptom and nearly one third (31.0%) reported ≥ 4 symptoms, while 23.1% reported 2–3, 17.1% reported 1, and 28.8% reported no symptoms (mean 2.54, median 2, range 0–13). Survivors of breast, female reproductive cancer, and leukemia/lymphoma were most likely to report ≥ 4 symptoms, whereas melanoma and male reproductive cancer survivors were most likely to report no symptoms (Fig. 2).

### Factors associated with symptom count

In adjusted, multivariable negative binomial regression analysis, as indicated by the rate ratio, the number of reported symptoms were significantly increased among those who were female (vs. male), were Hispanic ethnicity (vs. non-Hispanic white), had public insurance (vs. employer-sponsored), and received chemotherapy/radiation (vs. surgery only) by 44%, 21%, 49%, and 71%, respectively (Table 3).

**Table 3:** Associations of demographic and clinical characteristics with symptom count*

	Multivariable Analysis			
	*b*	SE	*p*	RR (95%CI)
Attained age	−0.02	0.01	**0.0074**	0.98 (0.07, 1.00)
Sex				
Male	(referent)			
Female	0.36	0.08	** < 0.001**	**1.44 (1.22, 1.69)**
Race and ethnicity				
Non-Hispanic White	(referent)	–	–	
Hispanic	0.19	0.09	**0.040**	**1.21 (1.01, 1.45)**
Non-Hispanic Asian	0.11	0.21	0.36	1.37 (0.91, 2.05)
Non-Hispanic Black	0.31	0.12	0.13	1.12 (0.88, 1.41)
Other or unknown	−0.20	0.29	0.48	0.82 (0.46, 1.44)
Neighborhood socioeconomic status				
Low	(referent)	–	–	
Low-middle	0.10	0.12	0.42	1.10 (0.87, 1.39)
Middle	0.01	0.13	0.96	1.01 (0.78, 1.29)
Middle-high	0.17	0.13	0.18	0.84 (0.65, 1.08)
High	−0.18	0.13	0.18	0.84 (0.65, 1.08)
Health insurance type				
Employer-sponsored	(referent)	–	–	
Public	0.40	0.10	** < 0.001**	**1.49 (1.22, 1.81)**
Individual	−0.05	0.14	0.69	0.95 (0.72, 1.24)
Other/Unknown	0.19	0.12	0.11	1.21 (0.96, 1.53)
No insurance	0.13	0.17	0.47	0.88 (0.63, 1.24)
Cancer stage				
Stage 0/1	(referent)	–	–	
Stage 2	0.08	0.11	0.43	1.09 (0.88, 1.34)
Stage 3	−0.03	0.13	0.84	1.03 (0.80, 1.32)
Stage 4	−0.10	0.19	0.59	0.90 (0.63, 1.31)
Unknown/not applicable	0.08	0.13	0.52	1.09 (0.84, 1.41)
Type of treatment				
Surgery only	(referent)	–	–	
Chemotherapy/no radiation	0.37	0.10	** < 0.001**	**1.44 (1.17, 1.77)**
Radiation/no chemotherapy	0.21	0.13	0.09	1.23 (0.96, 1.58)
Chemotherapy/radiation	0.54	0.11	** < 0.001**	**1.71 (1.38, 2.14)**

Abbreviations: *CI *confidence interval, *SE *standard error, *RR* rate ratio

*Sex-specific cancers excluded from this analysis

## Discussion

In this population-based study involving a sociodemographically diverse sample of over 1,000 long-term survivors of YA cancer, we report what is, to our knowledge, the first “landscape” analysis describing the types and prevalence of persistent or late-onset symptoms following multimodal therapy for varied cancer types. At nearly 10 years post diagnosis, more than two-thirds of participants were experiencing at least one clinically significant symptom, over half were experiencing 2 or more symptoms, and over one-third reported 4 or more, many of which map to major organ systems with potential for life-changing impact on function and quality of life. In contrast to the well-characterized population of young adults previously treated for childhood cancer [3, 4, 9, 20, 26], knowledge regarding adverse health outcomes among survivors of YA cancer is far more limited. The findings reported here indicate survivors of YA cancer similarly experience a substantial burden of clinically significant symptoms likely related to underlying late effects, an important addition to the nascent body of evidence describing this under-represented population. This information is essential for understanding and addressing their considerable impact on health status, quality of life, functionality, and achievement of milestones of emerging adulthood.

Due to the lack of published data on symptom burden from multi-diagnosis YA cohorts, comparison of our findings to other studies must be limited to single-cancer cohorts. Many of these studies come from heterogenous samples that include patients older than 39 at diagnosis, which likely influences study results. Allowing for this, the overall frequencies of reported symptoms are similar to our cohort, although specific patterns vary by cancer type and treatments received. For example, studies of early-onset (diagnosed < 50 years old) breast cancer survivors 2–10 years after treatment found that fatigue (25%–35%), cognitive dysfunction (20%–40%), and chronic pain (20%–30%) were the most common symptoms, and these were correlated with higher stage and multiple treatment modalities [27–31]. Among survivors of NHL diagnosed in both younger and older adults, cognitive impairment (28%–53%), fatigue (20%–37%) and sensory neuropathy (25%–33%) were the most frequent symptoms [32–34]. For both survivors of breast cancer and NHL, symptoms were increased among those who received combined treatment modalities, as observed in our cohort [27, 31, 34, 35]. Among long-term survivors of Hodgkin lymphoma, pain (40%–71%), fatigue (28%–45%), and shortness of breath (10%–43%) were the most common symptoms [36–40]. Among survivors of gynecological cancer 2–5 years post-treatment, fatigue (23%–61%), pain (20%–59%), and sensory neuropathy (13%–45%) were reported most commonly [41–43]. Unsurprisingly, focal symptoms such as urinary incontinence have been reported most often among survivors who received pelvic surgery for colorectal (14%–57%) and genitourinary (2%–51%) cancers [44–48]. Rates of self-reported urinary incontinence range from approximately 20% for early-onset colorectal cancer to 14%–44% for early-onset genitourinary cancer [46, 48]. The frequency of premature menopause as a self-reported condition in our study (approximately 30% of women) is within the broad range from existing studies that reported a medically confirmed diagnosis (5%–60%) [41, 49–51]. Our findings are consistent with the AYA HOPE study that noted 51% of participants reported 3 or more symptoms and reported significantly more fatigue, pain, and other health conditions compared with non-cancer controls [18, 19]. Given that sample was limited to survivors within 36 months of diagnosis, our findings suggest many symptoms persist years later.

Comparison of symptoms with non-cancer peers is similarly limited but generally indicates a substantially higher prevalence in our cohort. For example, in multiple studies fatigue has been reported by approximately 20% of young adults compared with 40% in our cohort [52–55]. Whereas symptoms of sensory neuropathy range from 1%–7% among young adults in general, 23% of our sample endorsed this symptom [56, 57]. Similarly, cognitive difficulty was endorsed by more than one third of our survivors, while in the general population of young adults, the prevalence of self-reported cognitive dysfunction outside of a diagnosis of depression has been estimated at about 6% [58]. Although breathing difficulty is not common among otherwise healthy young adults, it was reported by 18% of our participants, which is roughly twice the prevalence of asthma in the US (approximately 8% in both young adults aged 22–39 and older adults aged 35–64) [59, 60]. Thus, where comparisons can be made with non-cancer young adults, our participants reported higher symptom prevalence.

Equally striking is the implication of potentially disabling impact of these symptoms on emerging adult survivors. For the three most frequently endorsed symptoms among our participants, i.e., fatigue, altered appearance, and cognitive functioning, prior studies have documented their negative effects on physical functioning, psychological wellbeing, social participation, and occupational achievement [61–67]. Further, chronic symptoms are likely to increase over time in number, prevalence, and severity with the emergence of comorbidities related to aging [68, 69].

This current report of symptom burden complements two recent publications describing the prevalence of medically confirmed chronic health conditions in multi-diagnosis cohorts of long-term survivors of YA cancer [16, 17]. In one study, the most common medical conditions detected included cardiovascular disease, thyroid problems, diabetes, and chronic liver disease [16]. In another study, over half of participants had been diagnosed with more than one medical condition in the 5–20 years following cancer treatment, with the most common conditions involving the vision, digestive, endocrine, cardiovascular, and urinary systems [17]. These studies provide important information about medical outcomes in this population but alongside our findings highlight the importance of capturing complementary symptom information accessed only via patient report [70].

Within our sample, variations in symptom report were associated with certain demographic factors. Higher symptom counts were reported among Hispanics/Latinos and those with public and other/unknown insurance, despite controlling for other factors (including SES), suggesting that these groups may have higher unmet needs for symptom management. Prior studies indicate Hispanic cancer patients of all ages experience higher symptom burden due to a complex interaction between social determinants of health, healthcare access barriers, and communication challenges [71–74]. Because our participants were recruited from a population-based cancer registry, we enrolled those who are not receiving care or have more difficulty accessing care due to insurance status or other social determinants of health, resulting in poor symptom management. Even when excluding sex-specific symptoms, we found that females were independently more likely than males to report symptoms, a finding consistent with prior research observing sex differences in adverse event reporting [75, 76]. We did not detect an association between symptom count and cancer stage, likely due to enrichment for locoregional cancer using our sampling strategy.

Our findings must be viewed in the context of strengths and limitations of this study. A major strength includes population-based recruiting of a large, sociodemographically diverse sample of long-term survivors of YA cancer comprising multiple cancer types treated with multimodal therapy, features necessary for developing a comprehensive understanding of symptom burden and identifying patient subsets at greater risk. Limitations include lack of an internal control group of non-cancer peers for comparison of reported symptoms, as measuring symptom burden was not a primary aim of the parent study. Collapsing individual hematological and gynecological cancers into larger groups was necessary for analytic purposes but could obscure differences between diagnoses. Also, the questions used were not part of a validated symptom measure, leading to potential reporting bias and the inability to quantify symptom severity and frequency, limiting insight into their potential impact on daily life.

Validated symptom measures relevant to this population include the Patient Reported Outcome Measurement Information System (PROMIS) and the Patient-Reported Outcomes version of the CTCAE (PRO-CTCAE). PROMIS is a constellation of self-reported measures of global, physical, mental, and social health domains for adults in the general population and those living with a chronic condition [77]. Although there is no cancer-specific PROMIS module, the measures have been used extensively to study health outcomes in cancer patients and survivors [78]. PRO-CTCAE was developed by the National Cancer Institute to capture symptomatic toxicity by self-report [79]. An advantage of PRO-CTCAE for studies like ours is its inclusion of 78 specific symptoms and attributes rather than more general outcomes.

Nevertheless, these results provide a signal for a substantial burden of symptoms affecting long-term survivors of YA cancer. This has several implications for their future cancer care and research. First, it is clear this population should receive ongoing, medically appropriate survivorship care for early detection of late effects and provision of optimal supportive services. Related to this, both oncology and primary care clinicians caring for them should be vigilant for symptoms and prepared to treat or promptly refer these patients to appropriate specialists, as broadly implemented late effects screening has potential for improving their health trajectory. Second, additional research is urgently needed to better define symptom burden in this population with more optimally controlled studies using validated, age-appropriate symptom measures, as well as having access to medical records for more detailed information about cancer treatment exposures [77, 80]. Such studies will be essential for accurately characterizing symptoms and their prevalence, as well as establishing treatment-related risk factors, including prior relapse and subsequent primary cancer, and their possible association with co-morbidities in aging survivors. Finally, this inclusive understanding of symptom burden facilitates more effective advocacy by identifying unmet needs in aggregate, as well as in subsets defined by ethnicity and insurance status, all of which impact achievement of key life-stage goals in this vulnerable population.

## Supplementary Information

Below is the link to the electronic supplementary material.

Supplementary file1 (DOCX 577 KB)

Supplementary file2 (DOCX 21 KB)

Supplementary file3 (DOCX 19 KB)

## Data Availability

Aggregated, deidentified data may be shared on reasonable request to the corresponding author.

## Declarations

### Prior presentations

Presented in part at the 5th Global Adolescent and Young Adult Congress, Long Beach, CA, USA, June 20–23, 2023.
