# Supplementary material for: Symptom burden among long-term survivors of young adult cancer: a report from the Project Milestones cohort
Source: J Cancer Surviv. 2026 Feb 20;20(Suppl 2):1593–603. doi: 10.1007/s11764-026-01986-7 (PMC13082749; doi:10.1007/s11764-026-01986-7)
Supplement: Supplementary file 1 [file 11764_2026_1986_MOESM1_ESM.docx]

**Supplemental Figure 1. Symptom items from Project Milestones survey^[1]^**

1. Questions 7-22 and 27-32 included in analysis
