# Supplementary material for: Symptom burden among long-term survivors of young adult cancer: a report from the Project Milestones cohort
Source: J Cancer Surviv. 2026 Feb 20;20(Suppl 2):1593–603. doi: 10.1007/s11764-026-01986-7 (PMC13082749; doi:10.1007/s11764-026-01986-7)
Supplement: Supplementary file 2 [file 11764_2026_1986_MOESM2_ESM.docx]

**Supplemental Table 1. Comparison of survey responders and non-responders on cancer registry variables**

|  |  |  | **Test Statistic** | |
| --- | --- | --- | --- | --- |
| **Characteristic** | **Nonresponder**  (*n* = 3,229) | **Responder**  (*n* = 1,151) | ***χ^2^*** | ***p*** |
| Age at cancer diagnosis, years, No. (%) | |  | 1.58 | 0.66 |
| 21-25 | 579 (17.9) | 213 (18.5) |  |  |
| 26-29 | 674 (20.9) | 256 (22.2) |  |  |
| 30-34 | 933 (28.9) | 328 (28.5) |  |  |
| 35-39 | 1,043 (32.3) | 354 (30.8) |  |  |
| Attained age, years, No. (%) |  |  | 5.52 | 0.36 |
| 26-29 | 90 (2.8) | 32 (2.8) |  |  |
| 30-34 | 473 (14.6) | 191 (16.6) |  |  |
| 35-39 | 853 (26.4) | 301 (26.1) |  |  |
| 40-44 | 929 (28.8) | 343 (29.8) |  |  |
| 45-49 | 717 (22.2) | 237 (20.6) |  |  |
| 50-52 | 167 (5.2) | 47 (4.1) |  |  |
| Years since diagnosis, No. (%) | |  | 1.34 | 0.51 |
| 4-7 | 1,108 (34.3) | 405 (35.2) |  |  |
| 8-10 | 1,205 (37.3) | 440 (38.2) |  |  |
| 11-12 | 916 (28.4) | 306 (26.6) |  |  |
| Sex, No. (%) |  |  | 13.38 | **< .001** |
| Female | 2,067 (64.0) | 805 (70.0) |  |  |
| Male | 1,161 (36.0) | 345 (30.0) |  |  |
| Race and ethnicity, No. (%) |  |  | 49.30 | **< .001** |
| Hispanic | 1,374 (42.5) | 398 (34.6) |  |  |
| Non-Hispanic White | 1,162 (36.0) | 547 (47.5) |  |  |
| Non-Hispanic Asian | 393 (12.2) | 127 (11.0) |  |  |
| Non-Hispanic Black | 171 (5.3) | 42 (3.7) |  |  |
| Other or unknown | 129 (4.0) | 37 (3.2) |  |  |
| Neighborhood socioeconomic status, No. (%) | | | 12.27 | **0.015** |
| Low | 648 (20.1) | 183 (15.9) |  |  |
| Low-middle | 681 (21.1) | 231 (20.1) |  |  |
| Middle | 578 (17.9) | 217 (18.9) |  |  |
| Middle-high | 697 (21.6) | 272 (23.6) |  |  |
| High | 625 (19.4) | 248 (21.6) |  |  |
| Cancer diagnosis, No. (%) |  |  | 26.30 | **< .001** |
| Reproductive | 1,113 (34.5) | 332 (28.8) |  |  |
| Leukemia/Lymphoma | 837 (25.9) | 318 (27.6) |  |  |
| Thyroid | 333 (10.3) | 159 (13.8) |  |  |
| Breast | 287 (8.9) | 132 (11.5) |  |  |
| Colorectal | 333 (10.3) | 104 (9.0) |  |  |
| Melanoma | 326 (10.1) | 106 (9.2) |  |  |
| Cancer stage, No. (%) |  |  | 6.78 | 0.34 |
| 0 | 15 (0.5) | 2 (0.2) |  |  |
| I | 1,741 (53.9) | 621 (54.0) |  |  |
| II | 518 (16.0) | 203 (17.6) |  |  |
| III | 417 (12.9) | 139 (12.1) |  |  |
| IV | 146 (4.5) | 50 (4.3) |  |  |
| Not applicable | 223 (6.9) | 89 (7.7) |  |  |
| Unknown | 169 (5.2) | 47 (4.1) |  |  |
