# Supplementary material for: Symptom burden among long-term survivors of young adult cancer: a report from the Project Milestones cohort
Source: J Cancer Surviv. 2026 Feb 20;20(Suppl 2):1593–603. doi: 10.1007/s11764-026-01986-7 (PMC13082749; doi:10.1007/s11764-026-01986-7)
Supplement: Supplementary file 3 [file 11764_2026_1986_MOESM3_ESM.docx]

**Supplemental Table 2. Proportion of participants reporting symptoms by cancer type**

|  | **Cancer Type**  **n (%)^1^** | | | | | | | |
| --- | --- | --- | --- | --- | --- | --- | --- | --- |
| **Symptom^2^** |  | **Leukemia/Lymphoma**  **n = 289** | **Gynecologic**  **n = 221** | **Thyroid**  **n = 138** | **Breast**  **n = 106** | **Testicular**  **n = 90** | **Colorectal**  **n = 90** | **Melanoma**  **n= 91** |
| Fatigue |  | 117 (40.5) | 97 (43.9) | 71 (51.5) | 55 (48.1) | 25 (27.8) | 25 (27.8) | 18 (19.8) |
| Altered appearance |  | 122 (42.2) | 79 (35.8) | 47 (34.1) | 62 (58.5) | 25 (27.8) | 20 (22.2) | 12 (13.2) |
| Cognition problems |  | 107 (37.0) | 72 (32.6) | 41 (29.7) | 50 (47.2) | 16 (17.8) | 24 (26.7) | 15 (16.5) |
| General pain |  | 91 (31.5) | 66 (29.9) | 37 (26.8) | 40 (37.7) | 21 (23.3) | 17 (18.9) | 14 (15.4) |
| Sensory neuropathy |  | 76 (26.3) | 55 (24.9) | 35 (25.4) | 33 (31.1) | 15 (16.7) | 18 (20.0) | 11 (12.1) |
| Urinary incontinence |  | 35 (12.1) | 97 (43.9) | 20 (14.5) | 26 (24.5) | 8 (8.9) | 17 (18.9) | 2 (2.2) |
| Breathing difficulties |  | 70 (24.2) | 42 (19.0) | 27 (19.6) | 19 (17.9) | 12 (13.3) | 14 (15.6) | 5 (5.5) |
| Vision impairment |  | 49 (17.0) | 37 (16.7) | 19 (13.8) | 27 (25.5) | 7 (7.8) | 9 (10.0) | 4 (4.4) |
| Mobility problems |  | 45 (15.6) | 32 (14.5) | 16 (11.6) | 15 (14.2) | 12 (13.3) | 11 (12.2) | 6 (6.6) |
| Eating difficulties |  | 23 (8.0) | 18 (8.1) | 24 (17.4) | 11 (10.4) | 5 (5.6) | 4 (4.4) | 4 (4.4) |
| Hearing impairment |  | 22 (7.6) | 19 (8.6) | 8 (5.8) | 14 (13.2) | 15 (16.7) | 4 (4.4) | 2 (2.2) |
| Stool incontinence |  | 17 (5.9) | 21 (9.5) | 7 (5.1) | 4 (3.8) | 3 (3.3) | 23 (25.6) | 2 (2.2) |
| Limb difference |  | 6 (2.1) | 2 (0.9) | 1 (0.7) | 3 (2.8) | 2 (2.2) | 0 (0) | 3 (3.3) |
| Heart problem |  | 9 (3.1) | 4 (1.8) | 1 (0.7) | 0 (0) | 1 (1.1) | 1 (1.1) | 0 (0) |
| Ostomy |  | 2 (0.7) | 2 (0.9) | 1 (0.7) | 0 (0) | 2 (2.2) | 5 (5.6) | 0 (0) |
| Ureterostomy |  | 1 (0.4) | 1 (0.5) | 0 (0) | 0 (0) | 0 (0) | 0 (0) | 0 (0) |

^1^Proportion of participants within each cancer type.

^2^Symptoms limited to those that are non-sex-specific.
