# Supplementary material for: Symptom burden among long-term survivors of young adult cancer: a report from the Project Milestones cohort
Source: J Cancer Surviv. Author manuscript; Available in PMC 2026 Apr 15. (PMC13082749; doi:10.1007/s11764-026-01986-7)
Supplement: Supp_Fig1 [file NIHMS2159970-supplement-Supp_Fig1.docx]

**Supplemental Figure 1. Symptom items from Project Milestones survey^[[1]](#footnote-1)^**


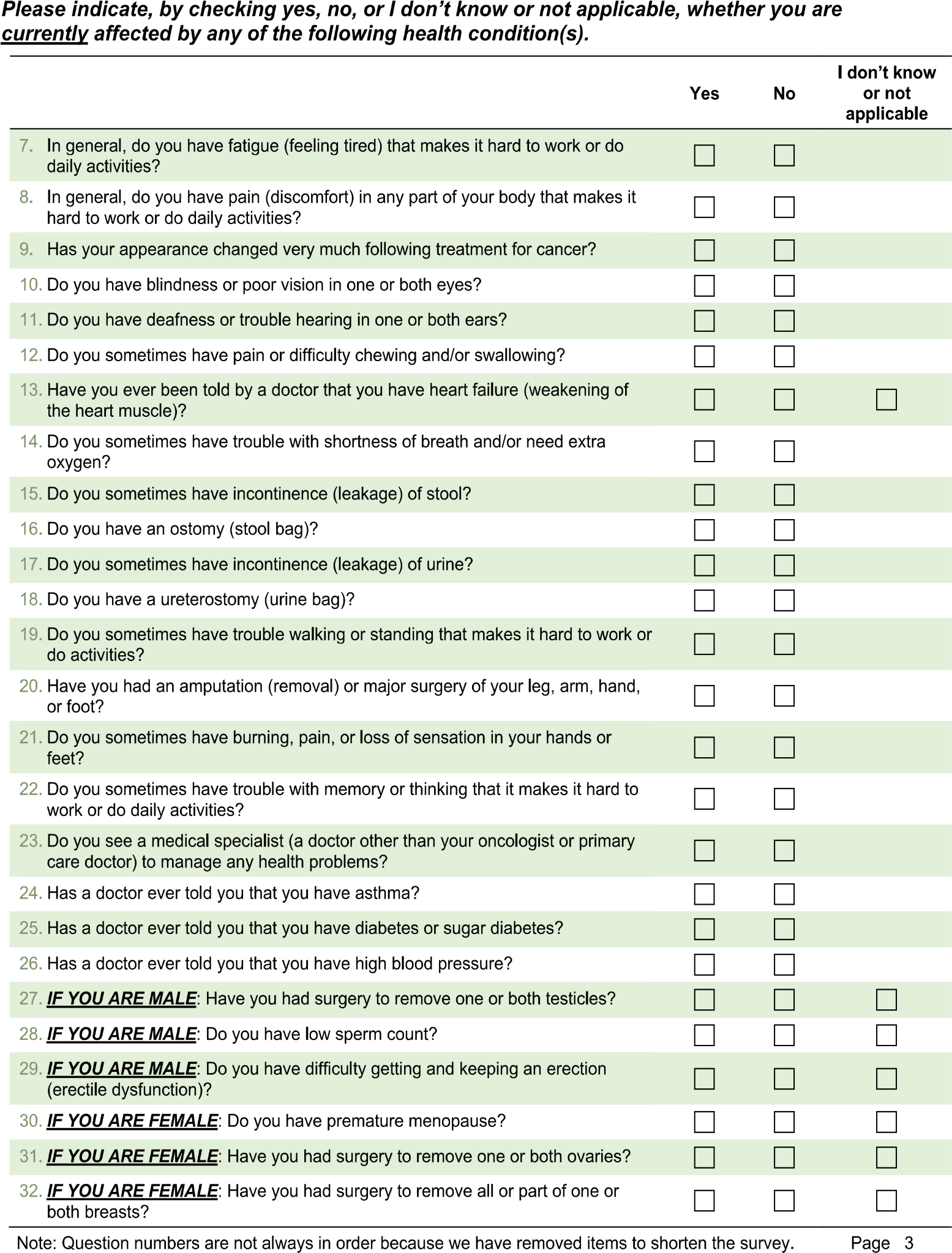


1. Questions 7-22 and 27-32 included in analysis [↑](#footnote-ref-1)
